# Impact of vaccine measures on the transmission dynamics of COVID-19

**DOI:** 10.1371/journal.pone.0290640

**Published:** 2023-08-25

**Authors:** Hua Liu, Xiaotao Han, Xiaofen Lin, Xinjie Zhu, Yumei Wei

**Affiliations:** 1 School of Mathematics and Computer Science, Northwest Minzu University, Lanzhou, Gansu, China; 2 School of Ecology and Environmental Sciences, Yunnan University, Kunming, Yunnan, China; 3 Experimental Teaching Department, Northwest Minzu University, Lanzhou, Gansu, China; Virginia Commonwealth University, UNITED STATES

## Abstract

In many nations, efforts to prevent and control COVID-19 have been significantly impeded by the SARS-CoV-2 virus ongoing mutation. The Omicron strain, a more recent and prevalent strain, has had more significant detrimental effects in countries worldwide. To investigate the impact of the Omicron BA.2 strain on vaccine efficacy, we proposed a model with vaccination and immunological decline in this research. Then, we fitted our model based on the number of daily new instances reported by the government in Jilin and Shanghai, China. We estimated the effective reproduction number *R*_*e*_ = 4.71 for the Jilin and *R*_*e*_ = 3.32 for Shanghai. Additionally, we do sensitivity analysis to identify the critical factors affecting the effective reproduction number *R*_*e*_. It was found that vaccination rate, effectiveness rate, and declining rate had a significant effect on *R*_*e*_. Further, we investigate the relevant parameter thresholds that make *R*_*e*_ lower than unity. Finally, rich numerical experiments were then carried out. We observed that even when vaccine efficiency was not high, increasing vaccination rates had a significant effect on early disease transmission, that limiting social distance was the most economical and rational measure to control the spread of disease, and that for a short period, reducing immune decline was not significant in curbing disease transmission.

## Introduction

From the initial SARS-CoV-2 virus infection in 2019 to the ongoing COVID-19 recurrent outbreak, more than two years have passed. The COVID-19 epidemic has created a serious threat to public health security and negatively impacted both social and economic progress and psychological health [1, 2]. Despite some stringent interventions (vaccination, city closures, community blockades, travel meltdowns, medical isolation, etc.) adopted by the Chinese government at the beginning of the phase outbreak, a cyclical epidemic character may still develop in mainland China due to the complexity of the virus’s mode of transmission [3, 4]. The number of fatalities and the risk of infection can be significantly decreased by non-pharmaceutical measures such as isolation and blocking [5, 6]. However, they frequently result in significant socioeconomic losses.

An efficient vaccine is one of the finest strategies to stop infectious diseases brought on by viruses. In a comparatively open setting, vaccination can dramatically lower the fatality rate and lessen the severity of infection [7]. Furthermore, Nicol Gozzi et al. showed that non-pharmacological measures during vaccination rollout are just as crucial to halting disease development [8, 9]. A press release from China’s National Health and Wellness Commission states that 1,234.54 million people nationwide—or 87.61% of the population—have completed all recommended vaccinations [10]. There are ongoing outbreaks even though the immunization rate is almost 90%. It’s because a variety of factors affect the effective of vaccines [11–15]. The effectiveness of the vaccine is influenced by several factors, including the nature of the virus and the vaccine itself. Alpha, Beta, Gamma, Delta, and Omicron (includes BA.1, BA.2, BA.3, BA.4, BA.5, and descendent lineages) are the main variation of concern (VOC) strains of SARS-CoV-2. Additionally, it contains circulating recombinant BA.1/BA.2 strains like XE) [16], of which the Omicron strain is the most recent version to be common in mainland China [17, 18]. One of the main obstacles to herd immunity is the ongoing development of viruses [19].

Vaccination rate and efficiency are important elements impacting vaccine effectiveness and are of research interest. Vaccines are one of the most effective ways to prevent and control infectious diseases. To characterize the immunization schedule, Manuel Adrian Acua-Zegarra et al. developed an optimal control problem with mixed constraints and ran simulations of vaccine efficacy, coverage, and induction. By simulating vaccine efficacy, coverage, vaccine induction, and natural immunity, a vaccination plan that reduces the burden of COVID-19 was developed [20]. When vaccine supplies are scarce, Yinggao Zhou et al. suggested a vaccination approach and came to the conclusion that the control technique should be changed to time-varying vaccination rates [21]. In order to create an optimal control problem for each aspect separately, Anuj Kumar et al. took into account a vaccine model with information induction and limited treatment. They discovered that the application of control strategies in combination lessened the severity of the disease burden and the associated financial burden [22]. Thompson et al. developed tools to estimate time-varying reproduction numbers characterizing the spread of infectious diseases, based on which control interventions during epidemics can be optimized [23]. The literature provides extensive information on more recent relevant investigations [24–31].

As a result of this wave of outbreaks brought on by the Omicron variety, we examine in this research the effects of various vaccination rates and efficacy rates on the control of the epidemic in mainland China. In Section 2, an ODEs model for the population-wide dissemination of COVID-19 is created while taking the vaccination rate and effectiveness rate into account. Some critical model parameters are fitted based on the actual number of reports in Section 3. In Section 4, the analytical formulas for the thresholds for the sensitivity analysis and effective reproduction number are obtained. An extensive numerical experiment is conducted in the next section. The final section is a discussion of our ongoing research.

## Mathematical model

Direct contact between a healthy person and a virus carrier, often known as direct transmission, is the main way that COVID-19 is spread [32]. Furthermore, in indirect transmission, healthy people can contract the virus by coming into touch with virus particles that are released into the air when an infected person sneezes, spits, or coughs [33]. Because SARS-CoV-2 viruses can live after being isolated from the host for hours or even days in the right circumstances, indirect transmission can occur [34]. The chance of contracting a virus through direct contact with an infected person is higher than the risk of contracting a virus from an object’s surface, according to research by Xin Zhang et al. [35]. Consequently, we solely considered the novel coronavirus’s direct transmission in the population.

Vaccines quickly cause people to develop vast numbers of antibodies in reaction to a subsequent viral invasion after entering the human body. Due to the complexity of the immune response process, vaccination efficacy is not always guaranteed [11]. The amount of antibodies in the body eventually diminishes as a result of the metabolic process of the individual, and this decline in antibody levels is constant across all populations, independent of the recipient’s gender, age, or history of chronic disease [12]. As a result, we defined the parameter *δ* described the immunological decline phenomenon.

When considering the dynamics of the spread of COVID-19 in a population, we divided the total population N into five components according to the individual’s status in the space of the disease: Susceptible (*S*), Exposed (*E*), Asymptomatic infected (*I*_*A*_), Symptomatic infected (*I*), and Recovered (*R*). After a period of time, some of those exposed over time develop confirmed symptoms of infection, while others remain asymptomatic. Over time, the asymptomatic infected either become symptomatic or clear the virus with the immune system or medication and become recovered. The symptomatic infected become recovered after medication. The immune level of recovered individuals declines over time until they become susceptible again.

According to national guidelines for the prevention and management of COVID-19 outbreaks, symptomatic patients are isolated medically as soon as they are identified. However, there is a risk of transmission through healthcare workers [36, 37]. We hypothesized that (1) exposed individuals carry the virus but are not infectious. Asymptomatic and symptomatic infected individuals have the ability to infect healthy individuals. (2) individuals who become recovered are at risk of reinfection [38, 39]. (3) Individuals have the potential for natural death at each stage of virus transmission. (4) Vaccinated individuals are those who have completed complete vaccination according to the current vaccine strategy. (5) There is no difference in the rate of immune decline for all individuals.

A point worth noting is that many researchers, when considering data based on actual cases, refer to the inverse of the rate of transmission from exposed to infected as the incubation period. However, there is often an error between the statistically obtained data and the true incubation period, due to the fact that many exposed individuals are classified as infected only after they have been diagnosed, but in fact, become infected before they are diagnosed. Therefore, when combining actual infection data, the inverse of the transmission rate from exposed to infected cannot simply be called the incubation period.

**Fig 1:**
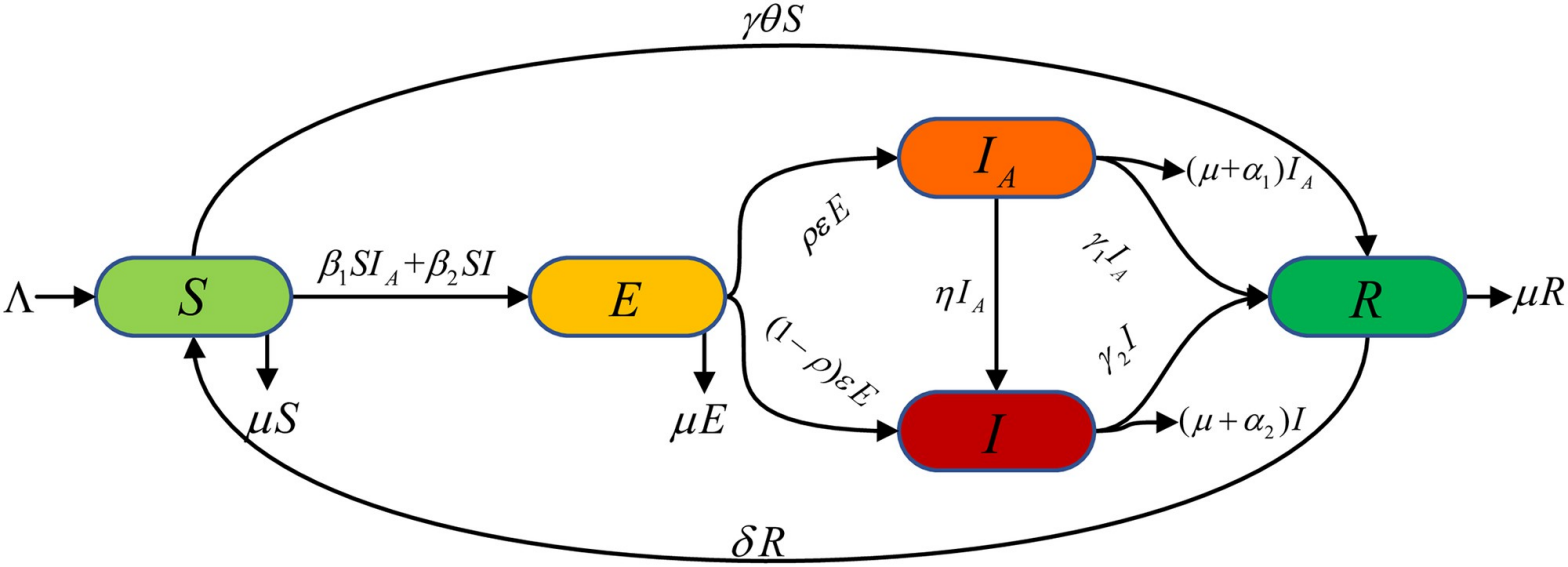
COVID-19 transmission pathways in the population.

Based on the previous description and assumptions, the following ODEs model was developed to simulate the transmission dynamics of COVID-19 in the population. Fig 1 shows the corresponding transmission path diagram.

$$\begin{array}{c} \left\{ \begin{array}{c} \frac{dS}{dt}=\Lambda +\delta R-\gamma \theta S-\beta_{1}SI_{A}-\beta_{2}SI-\mu S,\\ \frac{dE}{dt}=\beta_{1}SI_{A}+\beta_{2}SI-\left(\varepsilon +\mu \right)E,\\ \frac{dI_{A}}{dt}=\rho \varepsilon E-(\eta +\gamma_{1}+\alpha_{1}+\mu )I_{A},\\ \frac{dI}{dt}=\left(1-\rho \right)\varepsilon E+\eta I_{A}-(\gamma_{2}+\alpha_{2}+\mu )I,\\ \frac{dR}{dt}=\gamma \theta S+\gamma_{1}I_{A}+\gamma_{2}I-\left(\delta +\mu \right)R. \end{array}\right. \end{array}$$ (1)

where *N* = *S* + *E* + *I* + *I*_*A*_ + *R*. The initial condition for system (1) takes the form

$$\begin{array}{c} S\left(0\right)>0,E\left(0\right)\geq 0,I_{A}\left(0\right)\geq 0,I\left(0\right)\geq 0,R\left(0\right)\geq 0. \end{array}$$ (2)

Here Λ indicates the recruitment rate of the susceptible population. The immune level of recovered individuals declines at the rate of *δ*. *γ* denotes the vaccination rate. Parameter *θ* represents the vaccine efficiency rate, and *γθ* means the proportion of susceptible persons successfully vaccinated. *β*_1_ and *β*_2_ represent the exposure rate of susceptible persons with asymptomatic infections and symptomatic infections, respectively. According to the actual treatment principles, we assume *β*_1_ > *β*_2_. *ε* is the transmission rate per unit of time (day, in case of COVID-19) that exposed individuals become infectious, and *ρ* denotes the proportion of exposed persons becoming asymptomatic infections. Then 1 − *ρ* represents the exposed becomes symptomatic and asymptomatic infected become symptomatic at the rate of *η*. *γ*_1_ and *γ*_2_ denote the treatment rates of asymptomatic and symptomatic infections, respectively. Parameters *α*_1_ and *α*_2_ represent the causal mortality rates of asymptomatic and symptomatic infections. Similarly, we assume *α*_1_ < *α*_2_. *μ* represents the natural mortality rate.

## Data and parameter estimation

For this section, we compiled the number of confirmed COVID-19 cases reported in mainland China from March 1 to May 31, 2022. Using epidemic data from Shanghai and Jilin, we adapted the improved Markov chain Monte Carlo (MCMC) approach to fit the model’s parameters.

### Data sources

**Fig 2:**
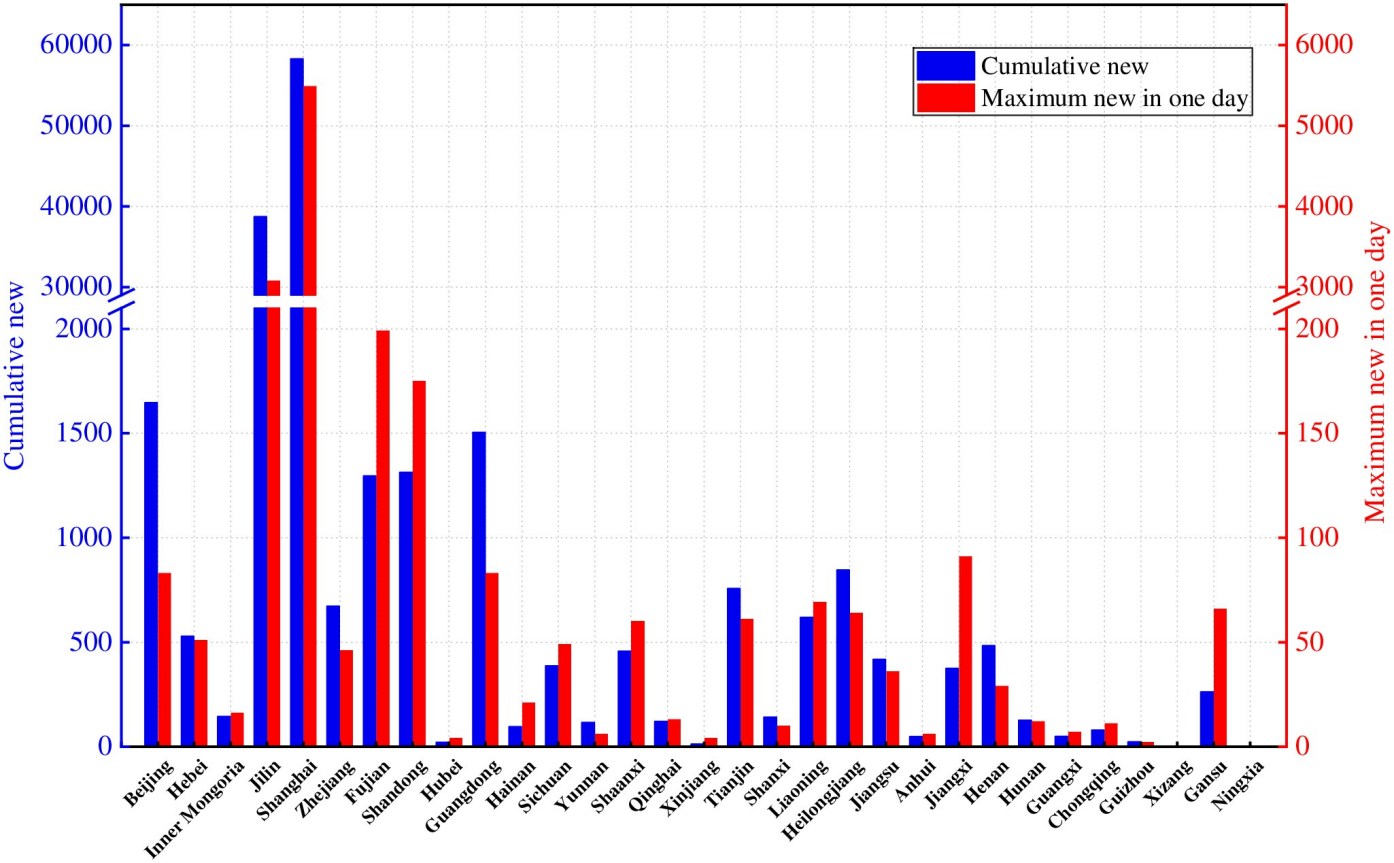
Cumulative number of infections by province and maximum number of infections in a single day. The blue column indicates the cumulative additions from March 1 to May 31, 2022 for each province. The red bar indicates the maximum value of single-day additions in each province from March 1 to May 31, 2022.

**Fig 3:**
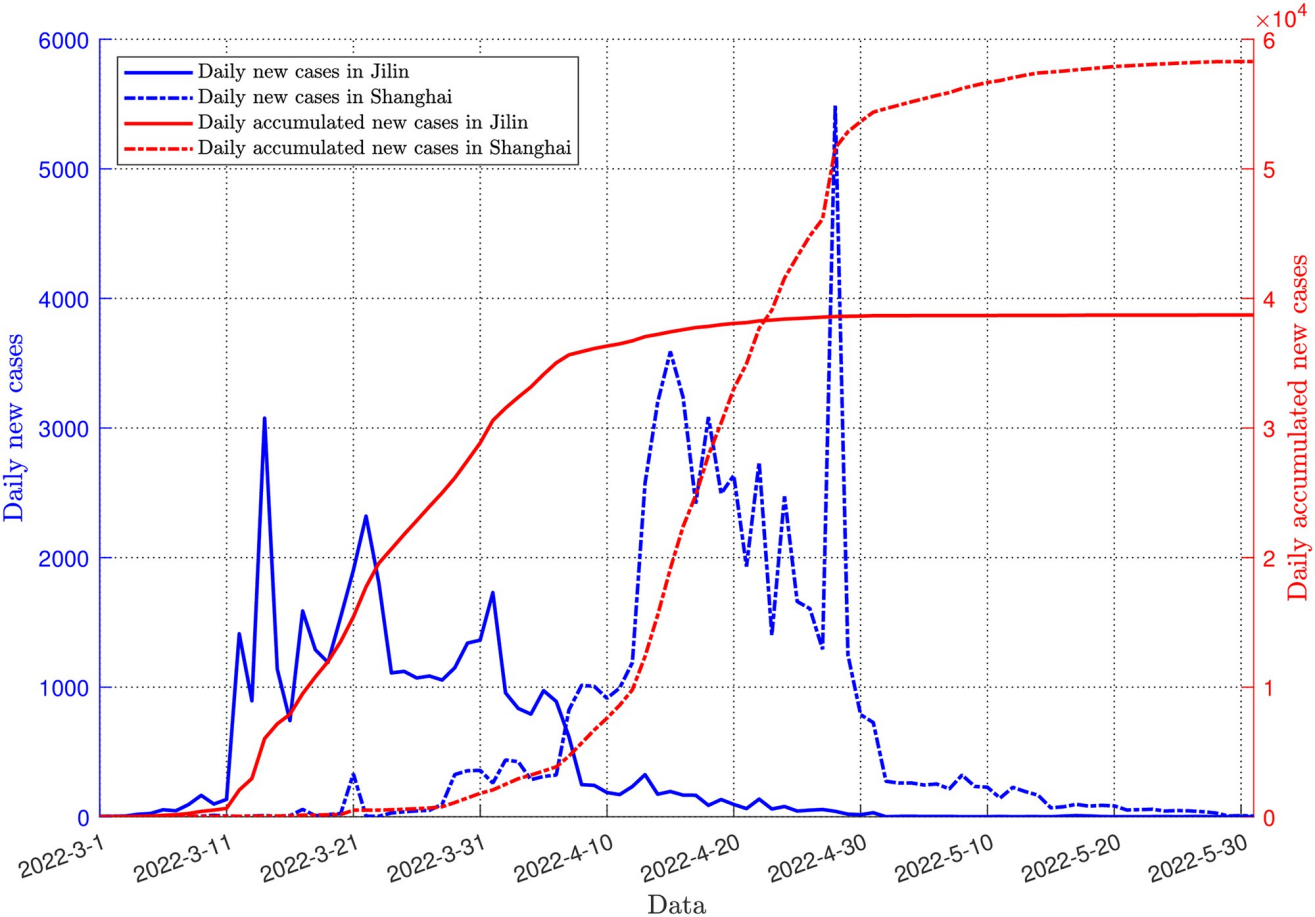
Trend of cumulative infections and daily new infections in Jilin and Shanghai. The blue solid line indicates the daily trend of new infections in Jilin, and the red solid line indicates the daily trend of new infections in Shanghai. The blue dotted line indicates the cumulative new infections in Jilin, and the red dotted line indicates the cumulative new infections in Shanghai.

We acquired epidemic data for each province (March 1-May 31) from the “COVID-19 Epidemic Prevention and Control Column” on each local government website in order to gain more precise surveillance data. Detailed data can be obtained from here (see S1 Appendix). The government department websites were updated daily with case data throughout the COVID-19 epidemic, which offers exceptional ease for collecting ongoing and comprehensive infection data. Fig 2 displays the total number of infections for each province on the Chinese mainland and the number of individuals with the highest number of new infections in a day from March 1 to May 31, 2022. The OmicronBA.2 strain most severely impacted Shanghai City and Jilin [40, 41]. Fig 3 displays the daily new and cumulative cases in the cities of Shanghai and Jilin. The number of illnesses rose quickly during the initial phase. The Omicron BA.2 strain is more contagious than the preceding strain, which is the first Omicron BA.2 strain pandemic in China. Due to the large numbers of infections in Shanghai and Jilin, using surveillance data from these two provinces to fit the model is realistic and representative.

The trend of the blue solid and dashed lines in Fig 3 shows that the daily number of new infections in Jilin Province fluctuates widely and the disease outbreak is relatively rapid, reaching a daily maximum of new infections in two weeks from March 1, suggesting that there may be occult transmission in Jilin Province. In contrast, the trend of daily new infections in Shanghai is relatively flat and more in line with the trend of natural transmission.

### Parameter estimation

By combining the Adaptive Metropolis (AM) algorithm with the Delayed Rejection (DR) method, Haario et al. created a DRAM-MCMC approach that is superior to the traditional MCMC method when without an excellent prior distribution [42, 43]. Reference [44] gives the DRAM-MCMC toolbox. Before the implementation of DRAM-MCMC, we obtained the values of some parameters based on statistical data. According to the statistical report of the national economic and social growth of Jilin in 2021 [45], the total population of Jilin is 23,753,700 people, and the average number of newborns born is $\tilde{\Lambda }=23753700\times 4.7‰/365$ per day, where 4.7‰ is the yearly birth rate [45]. Considering that births will be avoided during the epidemic and that newborns will receive enough protection from infection [46, 47], we assumed that the recruitment rate of the susceptible population was 5% of the birth rate. That is, the recruitment rate of the susceptible population in Jilin Province is $\Lambda =\tilde{\Lambda }\times 5\%=15.2935$ per day. The natural mortality rate in Jilin is obtained from the statistical bulletin on the development of health care in China in 2021 [48] as *μ* = 1/(78.2 × 365) per day, where 78.2 years is the national life expectancy per capita. From the Shanghai 2021 National Economic and Social Development Statistical Bulletin [49]. Shanghai has an annual resident population of 24,894,300 people, a natural mortality rate of *μ* = 1/(84.11 × 365) per day, and a life expectancy of 84.11 years. The yearly birth rate is 4.67‰, with an average birth rate of $\tilde{\Lambda }=24894300\times 4.67‰/365$ per day [49]. The recruitment rate of susceptible population in Shanghai was assumed to be $\Lambda =\tilde{\Lambda }\times 5\%=15.9255$ per day similarly.

**Fig 4:**
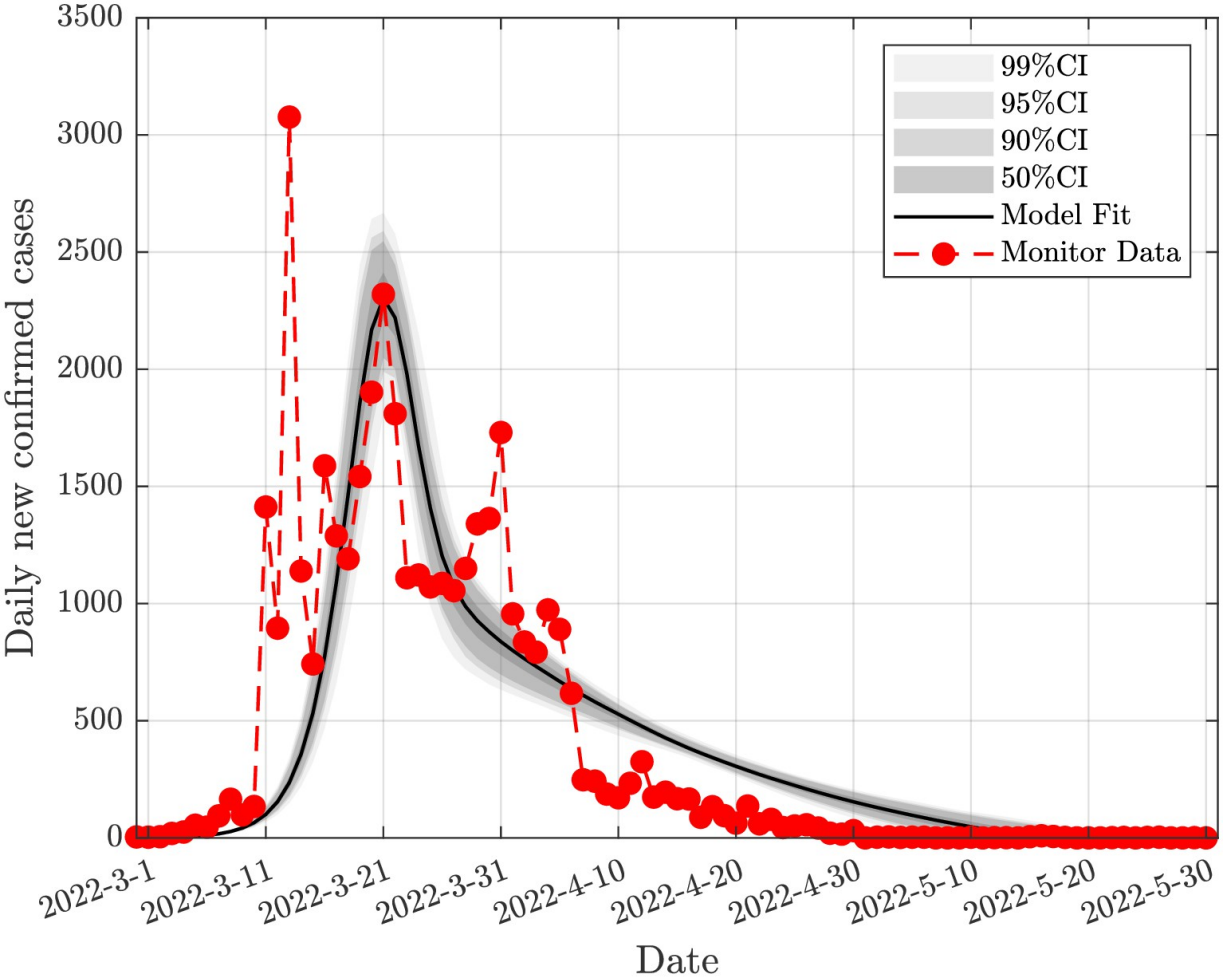
Model fitting based on daily new infection data in Jilin. The solid black line is the fitted value for Jilin, the gray area is the confidence interval, and the solid red dot is the observed data of Jilin.

**Fig 5:**
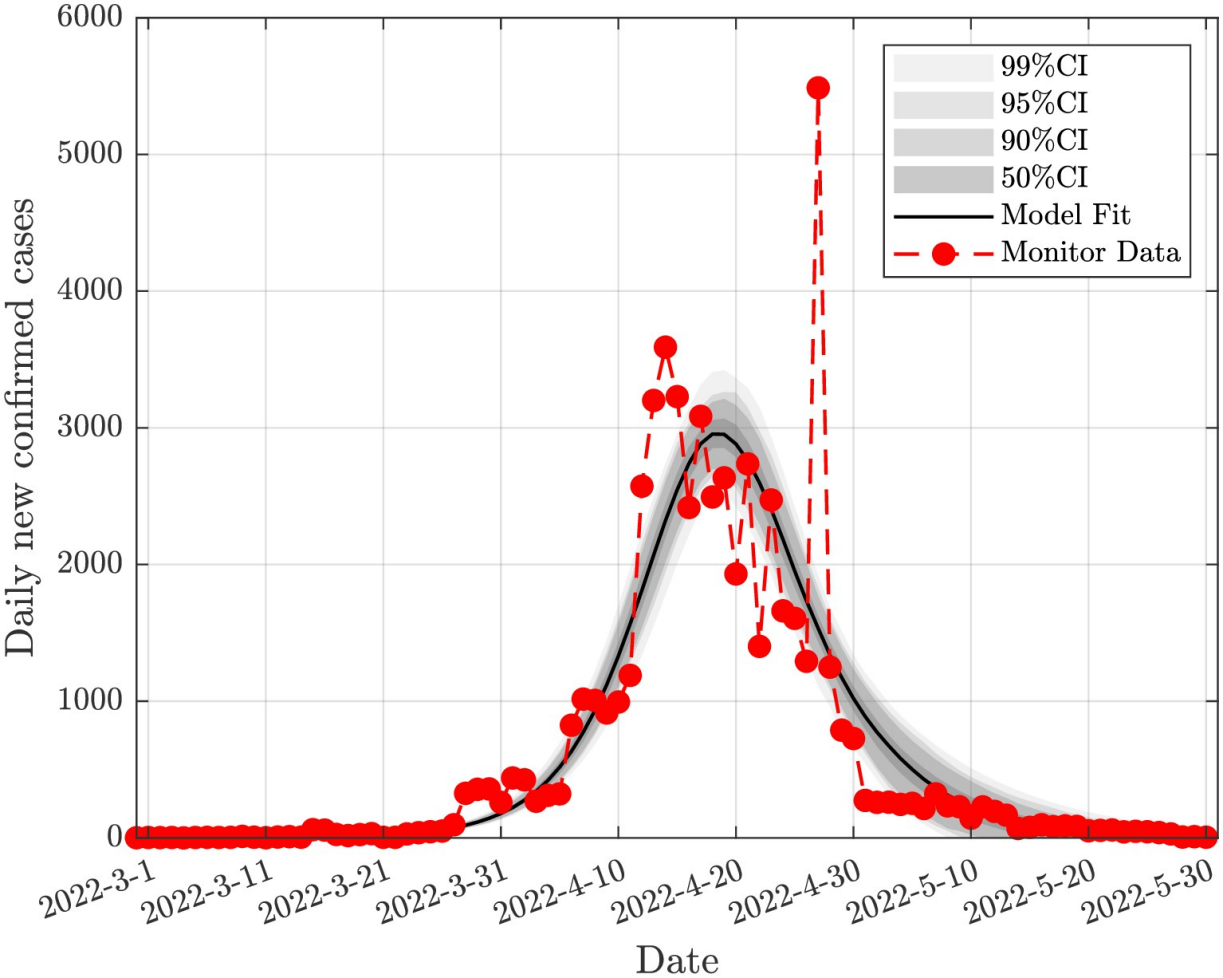
Model fitting based on daily new infection data in Shanghai. The solid black line is the fitted value for Shanghai, the gray area is the confidence interval, and the solid red dot is the observed data of Shanghai.

According to the relevant data released by the National Health Commission of the People’s Republic of China, as of February 25, 2022, 1,234,540,000 people have been fully vaccinated nationwide [50]. As of June 1, 2022, 1,256,857,000 people have been fully vaccinated nationwide [51]. From February 25, 2022 to June 1, 2022, the total number of people who have completed the full vaccination nationwide is 22,317,000 people. Therefore, the average vaccination rate is estimated to be *γ* = 0.0014. The prevented mortality rate for two doses of fire suppression vaccine is 66.8% [52] for inactivated vaccines used almost exclusively in mainland China. Omicron BA.2’s ability to bypass the immune system has yet to be fully understood [52]. Therefore, we chose a conservative vaccine effectiveness rate *θ* = 0.7. Table 1 displays the precise parameater values attained by DRAM-MCMC. The results of the fitting are shown in Figs 4 and 5, respectively, for Jilin and Shanghai.

**Table 1:** Parameters values and sources in system (1).

Parameters	Jilin	Shanghai	*Sources*
*γ*	0.0014	0.0014	*Assumed*
*θ*	0.7	0.7	[52]
Λ	15.2935	15.9255	*Estimated*
*μ*	3.50E-05	3.26E-05	*Estimated*
*β*_1_	4.82E-06	1.46E-06	*DRAM*-*MCMC*
*β*_2_	5.04E-08	5.35E-08	*DRAM*-*MCMC*
*δ*	6.13E-05	0.0046	*DRAM*-*MCMC*
*η*	0.0304	0.0803	*DRAM*-*MCMC*
*ε*	0.0062	0.0062	*DRAM*-*MCMC*
*γ*_1_	0.0008	0.0659	*DRAM*-*MCMC*
*γ*_2_	0.9670	0.7853	*DRAM*-*MCMC*
*α*_1_	6.30E-06	5.37E-08	*DRAM*-*MCMC*
*α*_2_	3.70E-05	5.19E-05	*DRAM*-*MCMC*
*ρ*	0.7857	0.8233	*DRAM*-*MCMC*
*S*(0)	23752596	24882546	*Estimated*
*E*(0)	1000	1000	*Estimated*
*I*_*A*_(0)	50	3	*Estimated*
*I*(0)	4	1	S1 Appendix
*R*(0)	14530851	15222894	*Estimated*

The parameter values in Table 1 show that the exposure rate of susceptible and asymptomatic infected individuals is greater in Jilin than in Shanghai. It is due to the occult transmission of this outbreak in Jilin province. In addition, the difference in population density and prevention and control measures resulted in different rates of transmission from exposed to infected persons in the two regions, but the differences were within acceptable limits.

## Effective reproduction number and sensitivity analysis

The effective reproduction number *R*_*e*_ is a cutoff value that we introduce in this subsection to measure disease transmission in the context of vaccination treatments. *R*_*e*_ is subjected to a global sensitivity analysis to identify the critical variables impacting the effective reproduction number. It is possible to acquire the parameter threshold that causes *R*_*e*_ to be equal to 1.

### Effective reproduction number

The basic reproduction number *R*_0_, a threshold value in epidemic systems generally, is used to decide whether a disease is extinct. The basic reproduction number, however, is only relevant when there are few sick people at the outset of an outbreak, and everyone else is vulnerable. We employ the effective reproduction number *R*_*e*_ here, a more appropriate criterion when interventions are present. The effective reproduction numbers illustrate the disease transmission under the intervention accurately.

First, a disease-free equilibrium point $H_{0}(\frac{\Lambda (\delta +\mu )}{\mu (\gamma \theta +\delta +\mu )},0,0,0,\frac{\Lambda \gamma \theta }{\mu (\gamma \theta +\delta +\mu )})$ for the system (1) can be easily obtained.

Further, we use the method of the next generation matrix (see S2 Appendix for the detailed procedure) [53] to get the effective reproduction number as

$$\begin{array}{c} R_{e}=\rho (FV^{-1})=\frac{(\beta_{1}h_{2}\rho +\beta_{2}\eta \rho +\left(1-\rho \right)\beta_{2}h_{1})\left(\delta +\mu \right)\Lambda \varepsilon }{\mu \left(\gamma \theta +\delta +\mu \right)\left(\varepsilon +\mu \right)h_{1}h_{2}} \end{array}$$ (3)

### Sensitivity analysis

**Fig 6:**
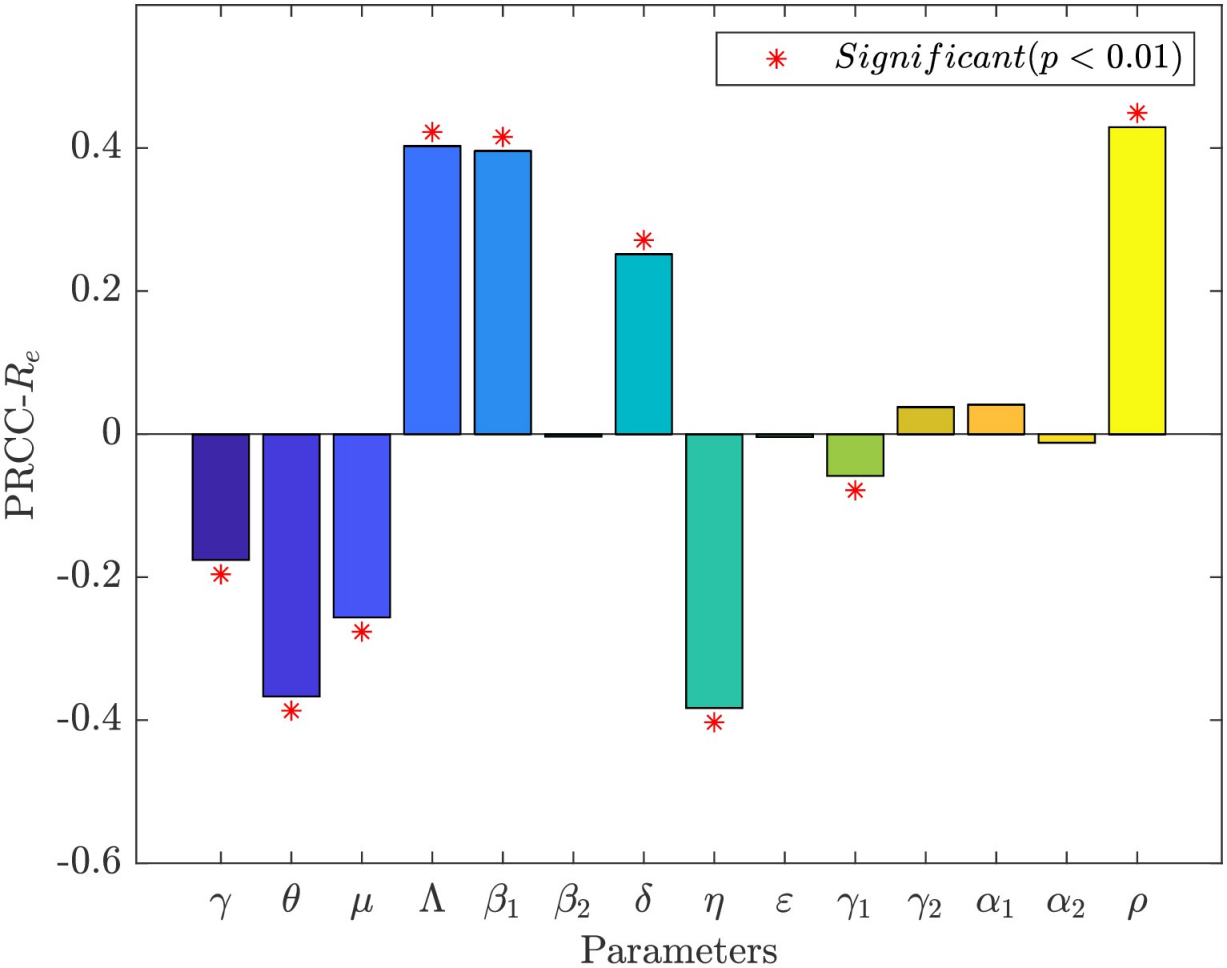
PRCC values and significance of model parameters for *R*_*e*_ in Jilin.

**Fig 7:**
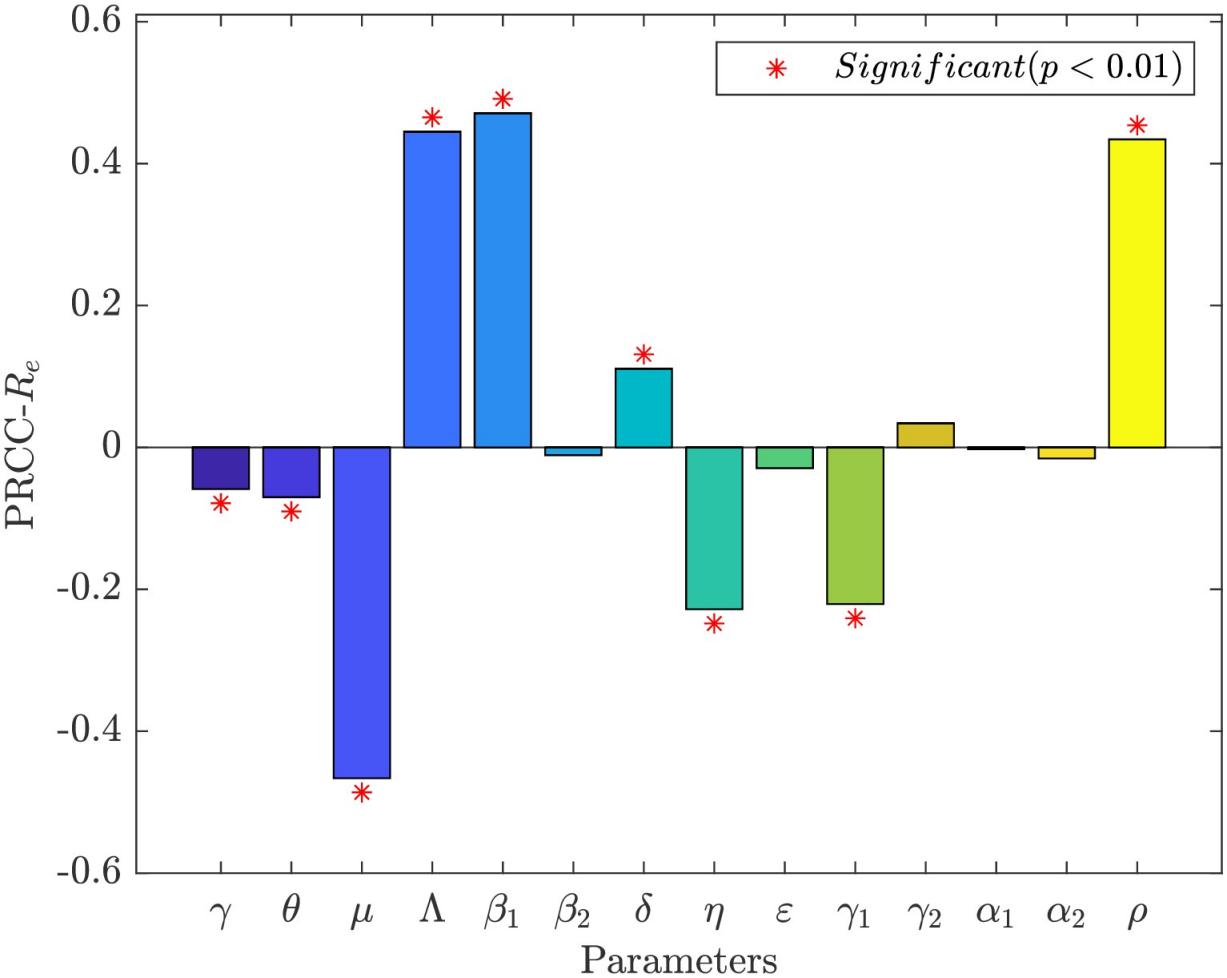
PRCC values and significance of model parameters for *R*_*e*_ in Shanghai.

In addition, we investigate the global sensitivity of the parameter of interest to one using the Latin Hypercube Sampling-Partial Rank Correlation Coefficient (LHS-PRCC) method [54]. We make the following assumption: that all parameters have a uniform distribution. The parameter range is 20% of the parameter estimates in Table 1, the sampling number is 1000, The results of the sensitivity analysis are shown in Figs 6 and 7, and the detailed values are shown in Table 2. The parameter affects Re positively if PRCC is positive. It has a detrimental impact otherwise. A more significant influence is indicated by a higher PRCC value. The effect of this parameter on *R*_*e*_ is negligible when the *p*-value is higher than 0.01 without changing the overall concept.

**Table 2:** PRCC values and *p*-values of parameters in system (1) for Jilin and Shanghai.

Parameters	Jilin		Shanghai	
	PRCC	*p*-values	PRCC	*p*-values
*γ*	−0.1760	*p* < 0.001	−0.0587	0.0087
*θ*	−0.3668	*p* < 0.001	−0.0702	0.0017
*μ*	−0.2563	*p* < 0.001	−0.4662	*p* < 0.001
Λ	0.4024	*p* < 0.001	0.4452	*p* < 0.001
*β*_1_	0.3955	*p* < 0.001	0.4713	*p* < 0.001
*β*_2_	−0.0036	0.8735	−0.0109	0.6263
*δ*	0.2513	*p* < 0.001	0.1112	*p* < 0.001
*η*	−0.3830	*p* < 0.001	−0.2282	*p* < 0.001
*ε*	−0.0040	0.8582	−0.0292	0.1913
*γ*_1_	−0.0585	0.0089	−0.2207	*p* < 0.001
*γ*_2_	0.0377	0.0917	0.0341	0.1269
*α*_1_	0.0412	0.0653	−0.0025	0.9111
*α*_2_	−0.0121	0.5879	−0.0156	0.4845
*ρ*	0.4291	*p* < 0.001	0.4341	*p* < 0.001

The parameters *γ*, *θ*, *μ*, *η*, and *γ*_1_ have a negative effect on the effective reproduction number *R*_*e*_ from Figs 6 and 7. That is, *R*_*e*_ decreases as the parameters increase. On the contrary, *R*_*e*_ increases with increasing parameters Λ, *β*_1_, *δ*, and *ρ*. In Jilin and Shanghai, increasing the rate of immune loss *δ* (natural and vaccine-induced immune loss) had a facilitative effect on disease transmission. Conversely, increasing the vaccination rate *γ* and vaccine effectiveness rate *θ* had a suppressive effect on disease transmission. Thus, it is further shown that system (1) can reflect the transmission of COVID-19 in the population to some extent realistically. Increasing the contact rate *β*_1_ has a facilitative effect on the spread of the disease. Social distance (wearing a mask, maintaining one-meter line, etc.) is still needed after vaccination to be more conducive to eradicating the disease. An increase in the proportion *ρ* of exposed individuals becoming asymptomatically infected contributes to the spread of the disease in the population. Therefore, increased Covid Test is needed at the beginning or during an outbreak to detect infected individuals as early as possible is essential to containing the spread of the disease. In addition, treatment for asymptomatic infected individuals is necessary, which will effectively lower the effective regeneration number.

### Critical threshold for *R*_*e*_ = 1

Let *L* = *γθ* and the parameter *L* denotes the percentage of successful vaccination. Assuming *R*_*e*_ = 1, we obtain the expression of L*, δ*, and $\beta_{1}^{*}$ as follows.

$$\begin{array}{cc} & L^{*}=\frac{\delta +\mu }{\mu h_{1}h_{2}\left(\varepsilon +\mu \right)}(\varepsilon (\Lambda (\beta_{2}\left(1-\rho \right)h_{1}+\rho (\beta_{1}h_{2}+\beta_{2}\eta ))-\mu h_{1}h_{2})-\mu^{2}h_{1}h_{2}) \end{array}$$ (4)

$$\begin{array}{cc} & \delta^{*}=\frac{\varepsilon \mu (h_{1}(\beta_{2}\Lambda \left(1-\rho \right)-h_{2}\left(L+\mu \right))+\rho \Lambda (\beta_{1}h_{2}+\beta_{2}\eta ))-\mu^{2}h_{1}h_{2}\left(L+\mu \right)}{\mu^{2}h_{1}h_{2}-\varepsilon (h_{1}(\beta_{2}\Lambda \left(1-\rho \right)-h_{2}\mu )+\rho \Lambda (\beta_{1}h_{2}+\beta_{2}\eta_{1}))} \end{array}$$ (5)

$$\begin{array}{cc} & \beta_{1}^{*}=\frac{((h_{2}\mu^{2}+(\left(L+\delta \right)h_{2}-\Lambda \beta_{2}\left(1-\rho \right))\mu -\beta_{2}\delta \Lambda \left(1-\rho \right))h_{1}-\beta_{2}\eta \rho \Lambda \left(\delta +\mu \right))\epsilon +h_{1}h_{2}\mu^{2}\left(L+\delta +\mu \right)}{h_{2}\rho \varepsilon \Lambda \left(\delta +\mu \right)} \end{array}$$ (6)

As a result, parameters satisfy one of *L* > L* or *δ* < δ* or $\beta_{1}^{*}<\beta_{1}^{*}$ can result in *R*_*e*_ < 1, which allows the disease to be eradicated more quickly while other parameters are held constant. Based on the parameters in Table 1, we estimated *R*_*e*_ = 4.71 ([3.61, 5.57], 90% CI) for Jilin and *R*_*e*_ = 3.32 ([3.30, 3.33], 90% CI) for Shanghai. The results were broadly similar to the mean effective reproduction number of 3.4 (range 0.88 to 9.4, median 2.8. IQR:2.03, 3.85) in reference [55].

## Numerical simulations

In order to more clearly see how vaccinations and immunizations impact disease transmission in our model, numerical experiments on the analytical results of the model using MATLAB (R2019b) are performed in this subsection.

**Fig 8:**
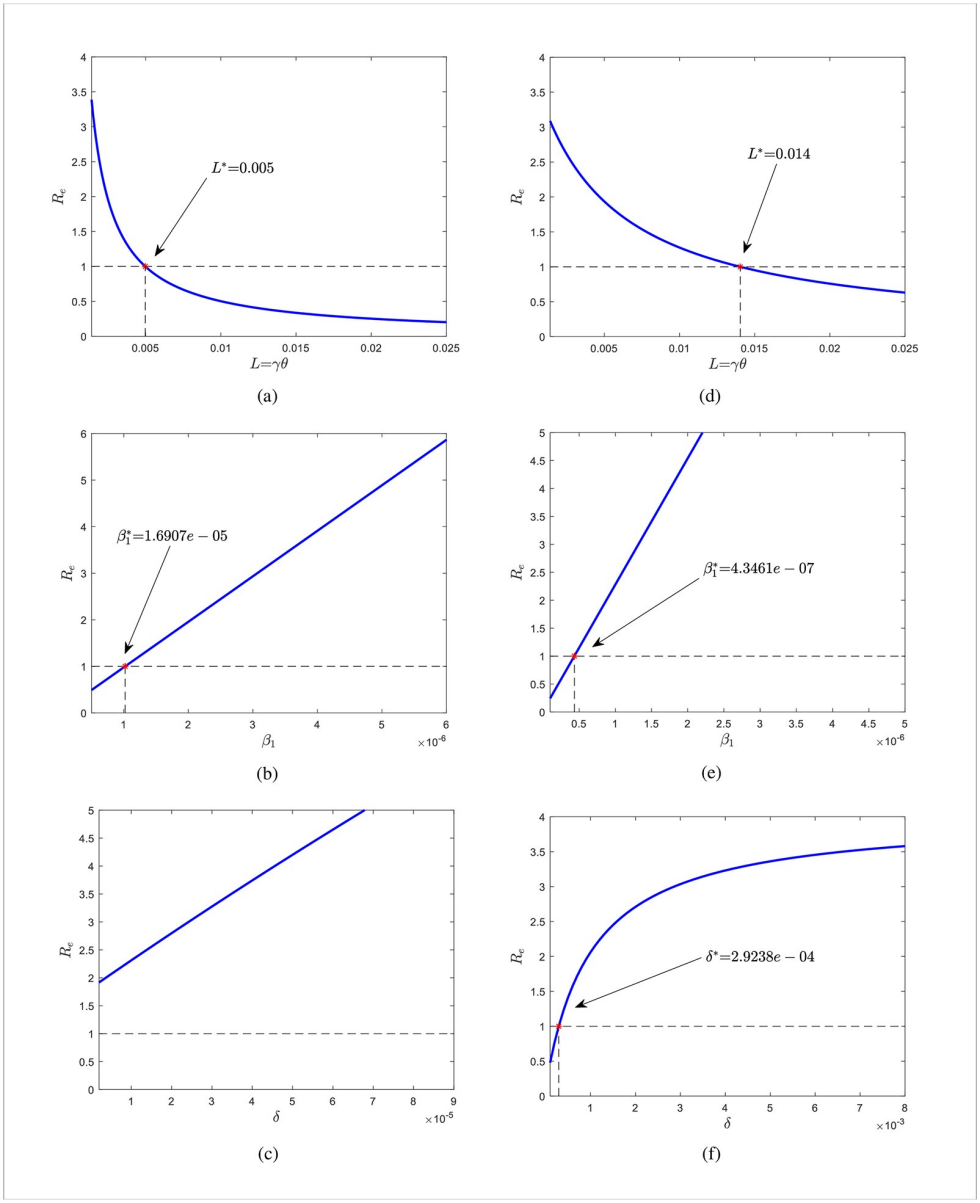
Evolution of *R_e_* with parameters for Jilin and Shanghai. (a)-(c) are the cases of Jilin and (d)-(f) are the cases of Shanghai.

**Fig 9:**
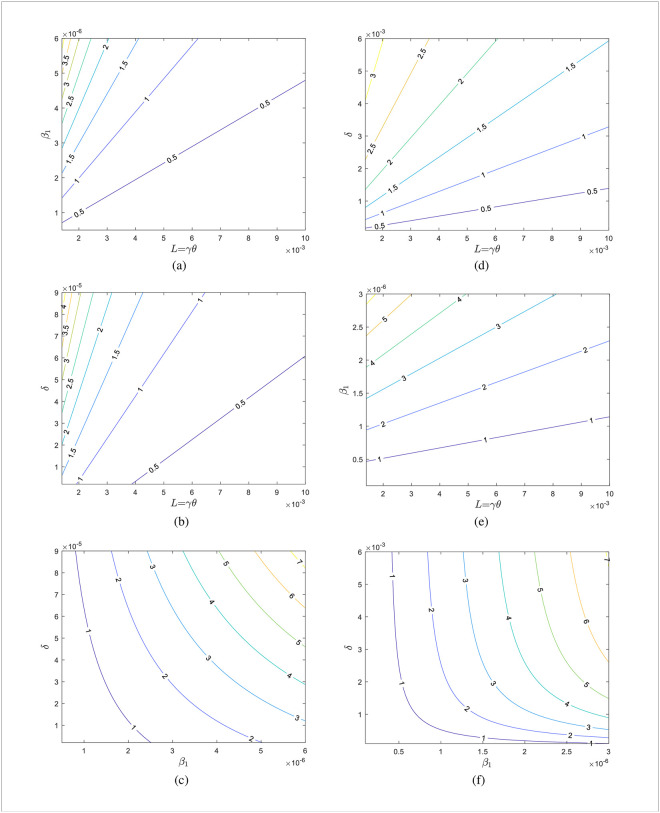
Contour plots of the effect of parameters on *R_e_* for Jilin and Shanghai. (a)-(c) are the cases of Jilin and (d)-(f) are the cases of Shanghai.

For Jilin, we take *L* ∈ [0.0014, 0.025], *β*_1_ ∈ [5.0E − 07, 6.0E − 06], and *δ* ∈ [2.0*e* − 06, 9.0*e* − 05] respectively. For Shanghai, we take *L* ∈ [0.0014, 0.025], *β*_1_ ∈ [1.0E − 07, 5.0E − 06], and *δ* ∈ [0.0001, 0.0.008] respectively. The other variables are all held constant. The impact of the parameters *L*, *β*_1_, and *δ* on *R*_*e*_ for Jilin is depicted in Fig 8(a)–8(c). Fig 8(d)–8(f) present the Shanghai case. The contour plots of each parameter’s impact on *R*_*e*_ are displayed in Fig 9(a)–9(c), respectively. The cases in Fig 9(d)–9(f) highlight the case of Shanghai.

According to Fig 8(a) and 8(d), more people need to be vaccinated on time for Re<1. For Jilin Province, the number of people successfully vaccinated per day needs to be greater than 0.5% of the total population, while for Shanghai it needs to be greater than 1.4%. Considering only the role of vaccines, more effort is required in Shanghai compared to Jilin Province. Vaccination rates also are affected by the amount of vaccine stockpiles and people’s willingness to get vaccinated. It is still a challenge to curb the development of the disease with vaccines alone. Fig 8(c) and 8(f) shows that booster vaccination alone does not curb disease transmission in Jilin province if other interventions remain unchanged, due to the presence of occult transmission in Jilin province. In contrast, booster shots can be given aggressively in Shanghai to reduce the rate of immune decline which is essential to curb the spread of the disease. *R*_*e*_ < 1 may be produced by altering both *L* and *β*_1_, *L* and *δ* in Fig 9(a), 9(b) and 9(d), 9(e), respectively. And this suggests that slowing disease transmission rates and immunological decline (due to both natural and vaccine-induced immunity) may be more successful in stopping the spread of the illness. Keeping a social distance is the best way to lower the transmission rate. Therefore, minimizing the vaccine-induced immunological decrease is the primary goal of slowing immune decline. And the most excellent way to slow immunological loss is to give booster doses of vaccines. Fig 9(c) and 9(d) illustrate the investigation into the combined impact of *β*_1_ and *δ* on *R*_*e*_. When *β*_1_ and *δ* meet particular criteria, there will be *R*_*e*_ < 1.

**Fig 10:**
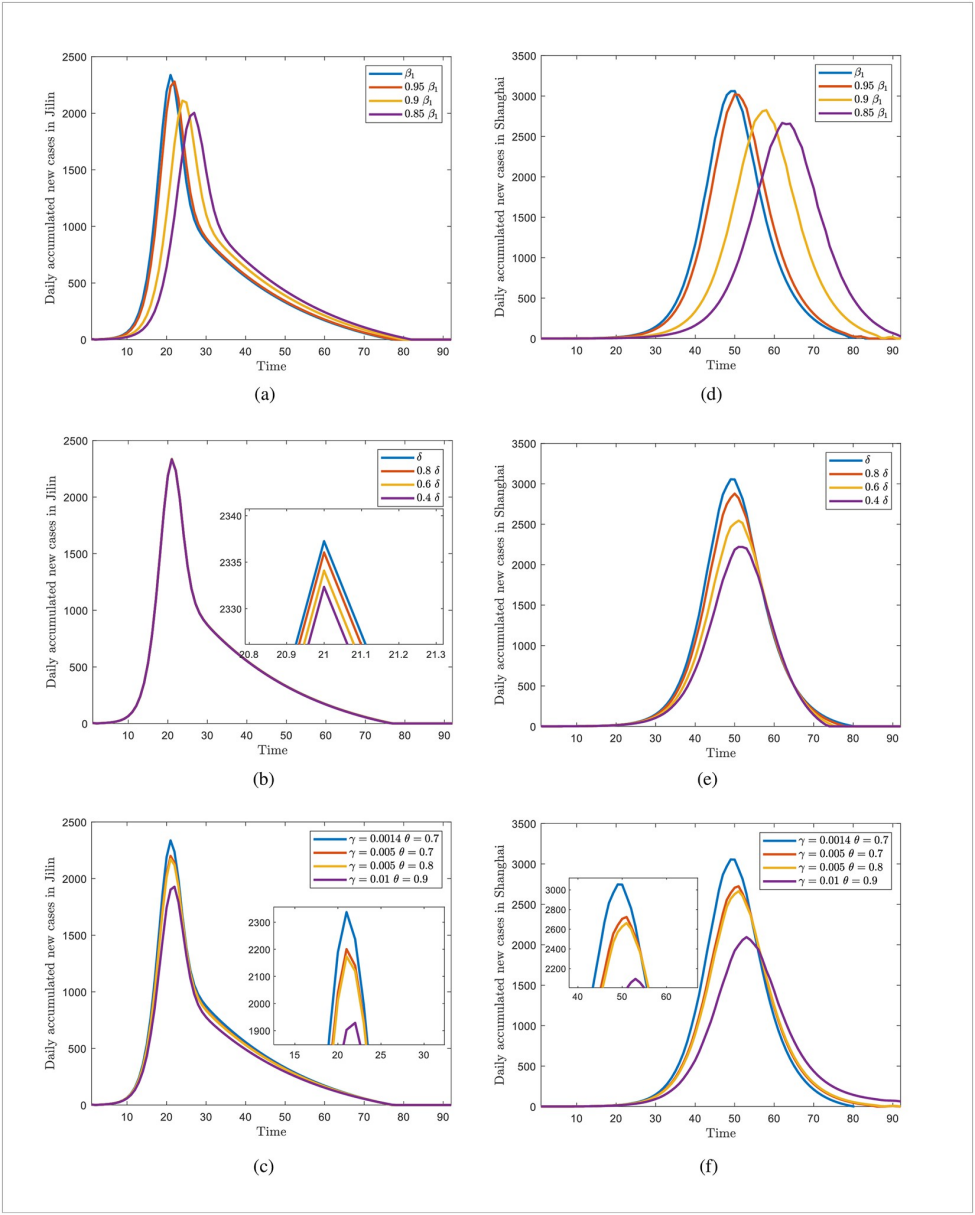
Effect of different intervention efforts on the final infection size in Jilin and Shanghai. (a)-(c) are the cases of Jilin and (d)-(f) are the cases of Shanghai.

Fig 10(a) and 10(d) demonstrate that reducing *β*_1_ reduces the peak number of new diagnoses each day and delays the peak’s arrival. This finding implies that timely vaccine boosters and protection against exposure during an epidemic’s early and emerging stages are essential because the delayed rise allows health management organizations more time to respond and create more effective control measures. Form Fig 10(b) and 10(e), it indicates that for Jilin Province, controlling disease transmission by reducing the rate of immune decline is not significant, probably because of the influence of occult transmission. In other words, the recession rate of immunization level has little effect on disease transmission in a short time. In contrast, reducing the immune decline rate can reduce and delay the peak number of infections in Shanghai. Fig 10(c) and 10(f) suggesting that improving vaccine efficiency does not have a significant impact on controlling disease transmission based on the majority of the population being vaccinated, but rather that vaccination rates should be increased. In conclusion, regardless of high or low vaccine efficacy, vaccination should be administered as soon as possible at the beginning of a disease outbreak.

## Conclusion and discussion

One of the critical strategies to stop epidemics from spreading is vaccination, which is affected by a variety of circumstances during implementation, such as people’s desire to receive shots and the supply of vaccinations. The effectiveness of the vaccination is also significantly influenced by them. The amount of research and development and the virus’s unpredictability frequently put a cap on vaccine effectiveness. Over time, the effect of vaccine-induced immunity fades, which also has an impact on how well vaccinations work to prevent illness. In order to investigate the effects of Omicron BA.2 on mainland China, we created an ODEs model, including vaccination and immunological decline.

The DRAM-MCMC approach was used to fit the model, and the observation data for the model was the daily number of new COVID-19 cases reported in Shanghai and Jilin, China, between March 1 and May 31, 2022. To further depict the disease’s prevalence in the context of therapies, we established the effective reproduction number Re. We calculated *R*_*e*_ = 4.71 ([3.61, 5.57], 90% CI) in Jilin and *R*_*e*_ = 3.32 ([3.30, 3.33], 90% CI) in Shanghai based on the results of parameter fitting. In order to identify the critical variables impacting *R*_*e*_, we conducted a global sensitivity analysis. According to research, *R*_*e*_ lowers when vaccination rates and vaccine efficacy rates rise.

In this paper, the transmission rates of exposed individuals becoming infected is also influenced by the population density of the two cities as well as control measures, in addition, some individuals need treatment after being infected, either asymptomatic or symptomatic, but the time to treatment is different between severe and mild cases, so it is more reasonable for us to consider the average treatment rates and average mortality rates. The size of the infected population in this epidemic differs in the two cities and the intensity of prevention and control measures, so it is more reasonable to use estimates to obtain transmission rates, treatment rates, and mortality rates.

In addition, decreasing the exposure and immune decline rates also reduced *R*_*e*_. We also found the threshold of the parameter that makes *R*_*e*_ < 1, and the results showed that the effect of vaccines alone could not completely contain the disease development in both Jilin and Shanghai. It is essential to increase the proportion of people successfully vaccinated and to improve personal protection to reduce the transmission rate. It is also necessary to call for more people to receive a booster dose of the vaccine.

A rich numerical experiment was also conducted to visualize the results of our study. *R*_*e*_ was also decreased by lowering the rates of immunological decline and exposure. However, for epidemics with occult transmission and rapid outbreaks but short duration, controlling disease progression by reducing the rate of immune decline is not as effective. Regardless of the type of disease epidemic, it is essential that as many people as possible are vaccinated in the early stages. We also determined the threshold of the parameter that makes *R*_*e*_ = 1. Lowering the transmission rate is critical to raise the number of persons who receive valid vaccinations and enhance personal protection. Calling more individuals to take a booster vaccination dose is also necessary. A detailed numerical experiment was also carried out to illustrate the findings of our investigation.

Our study also has two limitations; the exposure rate is not fixed and the vaccination rate is time-varying due to the different prevention and control policies at different stages of the disease. This requires more complex mathematical models to reflect the disease transmission process. Second, data on the number of asymptomatic infected and recovered persons and vaccination rates are not yet available, and vaccine efficacy rates are not publicly available. This poses a serious challenge for parameter estimation. In addition, qualitative and stability analysis of the ODE model was not performed in this paper. In future work, we hope to model different vaccination rate functions with the aim of finding a more realistic vaccination rate function. Thus, we can further improve the research related to the effect of vaccines on disease transmission.

## Supporting information

S1 Appendix New confirmed diagnosis data reported in mainland China from March 1-May 31, 2022. (XLSX)

S2 Appendix The procedure for calculating the effective regeneration number *R*_*e*_. (PDF)
