# Supplementary material for: Impact of vaccine measures on the transmission dynamics of COVID-19
Source: PLoS One. 2023 Aug 25;18(8):e0290640. doi: 10.1371/journal.pone.0290640 (PMC10464839; doi:10.1371/journal.pone.0290640)
Supplement: S2 Appendix — (PDF) [file pone.0290640.s002.pdf]

S2 Appendix

The regeneration and transfer matrices of system (1) are $\mathcal{F}$ and $\mathcal{V}$, respectively.

$$\mathcal{F}=\begin{bmatrix}\beta_1 SI_A+\beta_2 SI\\0\\0\\0\\0\end{bmatrix},\quad \mathcal{V}=\begin{bmatrix}(\varepsilon+\mu)E\\-\rho\varepsilon E+(\eta+\gamma_1+\alpha_1+\mu)I_A\\-(1-\rho)\varepsilon E-\eta I_A+(\gamma_2+\alpha_2+\mu)I\\-\Lambda-\delta R+\gamma S\theta+\beta_1 SI_A+\beta_2 SI+\mu S\\-\gamma S\theta-\gamma_1 I_e-\gamma_2 I+(\delta+\mu)R\end{bmatrix}$$

Then, by solving the Jacobi matrix at $H_0(\frac{\Lambda(\delta+\mu)}{\mu(\gamma\theta+\delta+\mu)},0,0,0,\frac{\Lambda\gamma\theta}{\mu(\gamma\theta+\delta+\mu)})$ for $\mathcal{F}$ and $\mathcal{V}$, we can obtain

$$D\mathcal{F}(H_0)=\begin{bmatrix}F&0\\0&0\end{bmatrix},\quad D\mathcal{V}(H_0)=\begin{bmatrix}V&0\\J_3&J_4\end{bmatrix}.$$

Let $h_1=\eta+\gamma_1+\alpha_1+\mu$, $h_2=\gamma_2+\alpha_2+\mu$, where the matrices F and V are as follows

$$F=\begin{bmatrix}0&\beta_1 S_0&\beta_2 S_0\\0&0&0\\0&0&0\end{bmatrix},\quad V=\begin{bmatrix}\varepsilon+\mu&0&0\\-\rho\varepsilon&h_1&0\\-(1-\rho)\varepsilon&-\eta&h_2\end{bmatrix}$$

$$J_3=\begin{bmatrix}0&\beta_1 S_0&\beta_2 S_0\\0&-\gamma_1&-\gamma_2\end{bmatrix},\quad J_4=\begin{bmatrix}\theta\gamma+\mu&-\delta\\-\theta\gamma&\delta+\mu\end{bmatrix}$$

then

$$V^{-1}=\begin{bmatrix}\frac{1}{\varepsilon+\mu}&0&0\\\frac{\rho\varepsilon}{(\varepsilon+\mu)h_1}&\frac{1}{h_1}&0\\\frac{\rho\eta\varepsilon+(1-\rho)\varepsilon h_1}{(\varepsilon+\mu)h_1h_2}&\frac{\eta}{h_1h_2}&\frac{1}{h_2}\end{bmatrix}$$

Therefore, the expression for the effective reproduction number can be obtained as follows

$$R_e=\rho(FV^{-1})=\frac{\rho\varepsilon\beta_1 S_0}{(\varepsilon+\mu)h_1}+\frac{(\rho\eta\varepsilon+(1-\rho)\varepsilon h_1)\beta_2 S_0}{(\varepsilon+\mu)h_1h_2}$$
$$=\frac{\left(\beta_1 h_2\rho+\beta_2\eta\rho+(1-\rho)\beta_2 h_1\right)(\delta+\mu)\Lambda\varepsilon}{\mu(\gamma\theta+\delta+\mu)(\varepsilon+\mu)h_1h_2}$$
